# Clapping-surpressed focal spikes in EEG may be unique for the patients with rett syndrome : a case report

**DOI:** 10.1186/s12883-016-0613-4

**Published:** 2016-06-13

**Authors:** Yudan Lv, Chang Liu, Mingchao Shi, Li Cui

**Affiliations:** Department of Neurology and Neuroscience center, First hospital of Jilin University, 71-XinminStreet, ChangChun, People’s Republic of China

**Keywords:** EEG, Rett syndrome, Centrotemporal spikes, Suppression by hand stereotypies

## Abstract

**Background:**

Rett syndrome is a severe neurodevelopmental disorder that primarily affects females. Typical features include a loss of purposeful hand skills, development of hand stereotypies, loss of spoken language, gait abnormalities, and acquired microcephaly. However, Rett syndrome hasn’t been recognized by clinical doctors at the early stage. So we need to find some special characters.

**Case presentation:**

We reported a Chinese case of Rett syndrome, exhibiting continuous centrotemporal spikes in EEG with paroxysmal suppression by hand stereotypies (hand clapping). The child, female, 4 years old, presented with a significant regression in her spoken language skills, hand stereotypies (hand clapping and hand wringing), a wider based gait with difficulties in balance, repeated abnormal behaviors (bruxism and head banging). With her clinical-history, Rett syndrome was suspected and genetic testing with mutation in MECP2 confirmed the diagnosis. Her EEG showed slow acticity in background and revealed a specific feature that continuous centrotemporal spikes can be suppressed by the repeated hand clapping. And when the hand stopped, the spikes reoccured again.

**Conclusions:**

This unique EEG signature has rarely been reported, which will expand the spectrum of EEG abnormalities in Rett syndrome.

## Background

Rett syndrome is classically characterized by early normal development for at least the first 6 months of life, followed by a period of regression and later recovery or Stagnation [1]. The main features of typical Rett syndrome include a partial or complete loss of purposeful hand skills, development of hand stereotypies, partial or complete loss of spoken language and gait abnormalities [2].

We reported one Chinese child diagnosed of Rett syndrome, with a specific electroencephalogram (EEG) feature that continuous centrotemporal spikes can be suppressed by the repeated hand clapping. The purpose of this report is to present a novel, rarely reported EEG characteristic, which may also aid diagnosis.

## Case presentaion

**Fig. 1:**
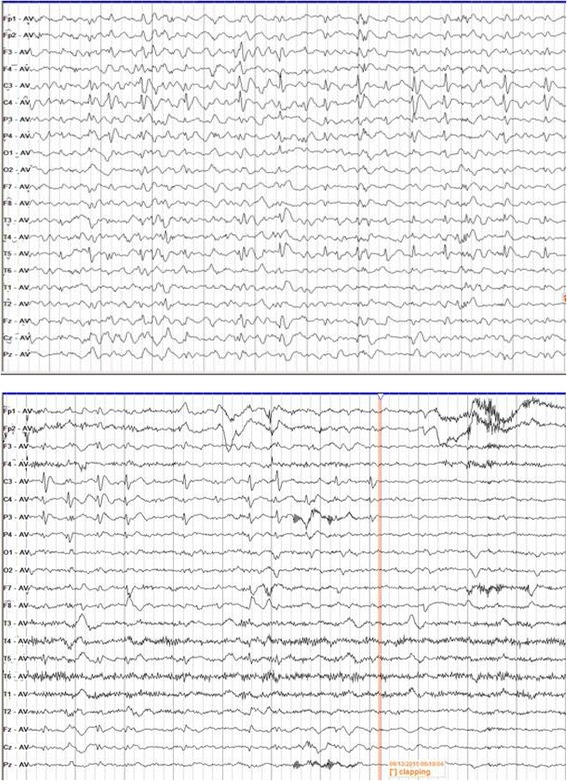
Continuous centrotemporal spikes in the EEG, and clapping-supressed focal spikes in EEG

A 4-year old female patient, presented with significant regression in her spoken language skills, hand stereotypies (hand clapping and hand wringing), a wider based gait with difficulties in balance, repeated abnormal behaviors (bruxism and head banging) for 2 years. In the early stage, her development was normal, her mother described that the little patient picked up objects around 5–6 months, sat unsupported at approximately 8–9 months and walked at around 14 months. Her first words of “ma, ma, ba, ba” were reported around 10–14 months, her simple words were reported around 18–20 months. However, at 24 months, a significant regression was noted in her spoken language skills, few words can be heard, and few communication with her parents. Subsequently, she was noted to develop hand stereotypies of hand clapping, hand wringing and have some difficulties in ordinary life, such as spoon-using, clothes-dressing. Then, she presented with a wider based gait and difficulties in balance. During this period, another abnormal behavior was observed, such as bruxism or head banging. With her clinical history, Rett syndrome was suspected. And in the next steps, EEG, magnetic resonance image (MRI) and genetic testing were given to confirm the diagnosis. Her MRI (brain) was normal. A 32-channel scalp EEG has been recorded for 24 h, and revealed mild diffuse θ\δ activity in the background. A lot of paroxysmal focal abnormal discharges of spike-slow were detected in the centrotemporal region, which persisted for a long time (Fig. 1). When we found such abnormal discharges, we have a detailed history-taking, but without any symptoms related to the epilepsy seizure. Under this situation, such discharges in EEG may not have any correlation with the epilepsy seizure. However, when the little patient did hand clapping or hand wringing, all the discharges in the EEG disappeared (Fig. 1), which was specific or unique EEG feature. This clapping-blocking response was similar to the Mu rhythm (normal physiolosical rhythm), which may indicated a inherent mechanism. Futhermore, the genetic testing with mutation in MECP2 confirmed the diagnosis.

## Discussion

We described a girl with Rett syndrome and unique EEG findings consisting of continuous centrotemporal spikes, which can be suppressed by the repeated hand clapping. Such an unusual EEG pattern has been rarely reported in individuals with Rett syndrome in recent years. However, early in 1990, Niedermeyer and Naida reported four cases of the Rett syndrome, where paroxysmal activity and especially spike discharges over the central region, could be blocked or attenuated by passive finger movements. Such blocking response are similar to our case and are also in the support of our findings. Besides this, Niedermeyer and Naida also presumed that the phenomenon found in RS is based on dysfunction rather than on structural impairment [3].

Rett syndrome often follow such four stages proposed: Normal development (0–6 months); Stage 1 (6–12 months), decreased head growth, delays in gross motor skills, loss of hand skills, less eye contact, social interaction and interest in toys; Stage2 (18 months–3 years), autistic features, intellectual disability, decreased head growth, respiratory abnormalities, hand stereotypies, motor dysfunction; Stage 3 (2–10 years), seizures are usually prominent, autonomic dysfunction; Stage 4 (≥10 years), loss of mobility, dystonia, scoliosis [2, 4].

The emphasis on the correlation of clinical and EEG findings should be clarified. The EEG findings often assume stereotypical patterns that similarly progress through the four clinical stages of the disease [5–7]. The most common EEG findings are summarized as follows: Stage1, normal, or slowing posterior background rhythm; Stage2, focal spikes in the centrotemporal regions, absent sleep spindles; Stage3, bilaterally synchronous delta activity and generalized spike discharges; Stage 4, slowing background activity, multifocal spikes in the waking, generalized spike activity during sleep, monorhythmic generalized or frontal-central slow theta (3–6 Hz) activity. Although an attempt to associate clinical staging with EEG abnormalities, the EEG changes are not specific to make a diagnosis of Rett syndrome. However, in our report, we found an special, or unique EEG signature that continuous centrotemporal spikes can be suppressed by the repeated hand clapping, once the hand stereotypies stopped, the spikes reoccured. This EEG feature has rarely been reported, which will expand the spectrum of electroencephalography abnormalities in Rett syndrome.

Additionally, Mu rhythm in EEG falls between 8 and 13 Hz, and is recorded from electrodes over the central area such as sensorimotor cortex, which suggests desynchronization of the underlying cell [8]. Both execution of actions, and passive or active movements have been found to block the activity of Mu rhythm [9]. Such blocking-response seems much more similar to RS. Furthermore, Jaime A Pineda [10, 11] argued that mirror neuron in Sensorimotor cortex played a critical role in the generation of Mu rhythm. Whether the underlying cells of mirror neurons involved in the blocking-response in RS, should be discussed in future research.

## Conclusion

The clapping-surpressed EEG pattern has rarely been described in Rett syndrome, which may provide clues for clinicians to the ultimate diagnosis. It remains to be determined whether this EEG pattern can also be encountered in other individuals with Rett syndrome. So we reported this pattern firstly, then hope our colleagues can pay attention to it.

### Aabbreviations

EEG, electroencephalogram; MRI, magnetic resonance image.

## References

[1] Dolce A, Ben-Zeev B, Naidu S, Kssoff E (2013). Rett syndrome and epilepsy: an update for child neurologists. Pediatr Neurol.

[2] Neul JL, Kaufmann WE, Glaze DG (2010). Rett syndrome: revised diagnostic criteria and nomenclature. Ann Neurol.

[3] Trauner DA, Haas RH (1987). Electroencephalographic abnormalities in rett syndrome. Pediatr Neurol.

[4] Hagberg B (2002). Clinical manifestations and stages of rett syndrome. Ment Retard Dev Disabil Res Rev.

[5] Hagberg B, Witt-Engerstrom I (1986). Rett syndrome: a suggested staging system for describing impairment profile with increasing age towards adolescence. Am J Med Genet.

[6] Niedermeyer E, Naidu SB, Plate C (1997). Unusual EEG theta rhythms over central region in rett syndrome: considerations of the underlying dysfunction. Clin Electroencephalogr.

[7] Niedermeyer E, Rett A, Renner H, Murphy M, Naidu S (1986). Rett syndrome and the electroencephalogram. Am J Med Genet Suppl.

[8] Niedermeyer E, Naidu S (1990). Further EEG observations in children with the rett syndrome. Brain Dev.

[9] Pfurtscheller G, Neuper C, Andrew C, Edlinger A (1997). Foot and hand area mu rhythms. Int J Psychophysiol.

[10] Chatrian G, Petersen M, Lazarte J (1959). The blocking of the rolandic wicket rhythm and some central changes related to movement. Electroencephalogr Clin Neurophysiol Suppl.

[11] Jaime A (2008). Pineda. Sensorimotor cortex as a critical component of an 'extended' mirror neuron system: does it solve the development, correspondence, and control problems in mirroring?. Behav Brain Funct.

